# When Trauma Crosses Generations: Mechanisms, Clinical Patterns and Therapeutic Implications of Transgenerational Trauma—A Systematic Review

**DOI:** 10.3390/cells15070609

**Published:** 2026-03-30

**Authors:** Oliwia Froń, Kamila Chwesiuk, Dominika Jabłonka, Agnieszka Kułak-Bejda

**Affiliations:** Department of Psychiatry, Medical University of Bialystok, pl. Wołodyjowskiego 2, 15-272 Białystok, Poland; 39754@student.umb.edu.pl (O.F.); 37059@student.umb.edu.pl (K.C.); agnieszka.kulak-bejda@umb.edu.pl (A.K.-B.)

**Keywords:** transgenerational trauma, transgenerational epigenetics, transgenerational inheritance, post-traumatic stress disorder, maternal stress, prenatal stress

## Abstract

Background: Transgenerational trauma (TT)/Intergenerational trauma (IT) is the transmission of the effects of traumatic experiences of parents to their children, who have not themselves experienced traumatic events. This transmission occurs through neurobiological and metabolic changes and the environment in which they were raised. Methods: A systematic review was conducted in accordance with the PRISMA 2020 guidelines. PubMed and Google Scholar databases were searched from 2005 to 2025. Studies focusing on adult offspring, exposure to ancestral trauma, biological markers (DNA methylation, cortisol), and psychological outcomes were included. Results: The main study results are as follows: identification of sex-specific DNA methylation patterns in the *NR3C1* gene and accelerated biological aging (GrimAge) in offspring; role of parental reflective functioning (PRF) and impaired mentalization as major psychological channels of trauma transmission; and evidence confirming the impact on three generations, manifested by treatment-resistant depressive disorders, anxiety, and neuroendocrine dysregulation (low cortisol levels). Conclusions: This article highlights the intergenerational impact of trauma and highlights its epigenetic significance. The primary goal was to explore universal epigenetic mechanisms. Early understanding of ancestral history is crucial for personalized psychiatric care.

## 1. Introduction

In psychology, trauma is an acute emotional reaction caused by a difficult experience, most often accompanied by a threat to the person’s life or their loved ones [1]. When it involves several traumatic experiences, it can, after some time, convert into complex post-traumatic stress disorder (CPTSD) [2]. In this case, we are talking about post-traumatic stress disorder (PTSD). Symptoms include reliving the traumatic event or events in the present in the form of intrusive thoughts, flashbacks and nightmares. Avoiding places, activities or people associated with the event. Seeing danger in everyday situations, excessive vigilance [1,3]. It can last for a minimum one month, which can lead to a decline in functioning [3,4]. Transgenerational trauma is associated with clinically meaningful manifestations in offspring across the lifespan, with symptom expression varying according to developmental stage [5,6]. In adult offspring, transgenerational trauma is reflected in increased vulnerability to post-traumatic stress symptoms, with evidence of sex-specific patterns of symptom expression [5,7]. In childhood, the impact of parental trauma is primarily expressed through behavioral and psychosocial adjustment difficulties rather than discrete psychiatric diagnoses [8,9]. Epigenetics is the science of genomic modifications that do not affect DNA sequences [10]. These modifications arise as a result of environmental interactions and can regulate gene expression in individuals who, in our case, have experienced trauma and their offspring [5,10]. Trauma can profoundly affect health, and early-life adversity may become biologically embedded, influencing health outcomes decades later [8]. One of the key pathways is DNA methylation—an epigenetic mechanism involving the addition of methyl groups to cytosine-guanine dinucleotide (CpG) sites, which can regulate gene expression and induce long-term biological effects [5,11,12]. DNA methylation does not alter the DNA sequence but determines how a given gene is expressed. In response to severe environmental stress, the body can mute genes responsible for stress resistance, resulting in subsequent generations having a modified stress response. However, the long-term effects of trauma can be more widespread, affecting not only mental but also physical health [10,13,14]. Early developmental manifestations of transgenerational trauma are likely observed in emotional regulation and the functioning of the parent–child relationship [9,15]. Transgenerational trauma is not diagnosed as a separate condition. Its presence is inferred based on a developmentally sensitive, transgenerational assessment of exposure to parental trauma and offspring functioning [1,6]. The aim of our systematic review was to assess the extent of intergenerational trauma transmission, identify biological and relational markers of transgenerational trauma, and explore a universal epigenetic mechanism for trauma inheritance. Trauma-informed and relationship-based interventions represent a clinically relevant strategy for mitigating vulnerability to transgenerational trauma across developmental stages.

## 2. Materials and Methods

**Figure 1:**
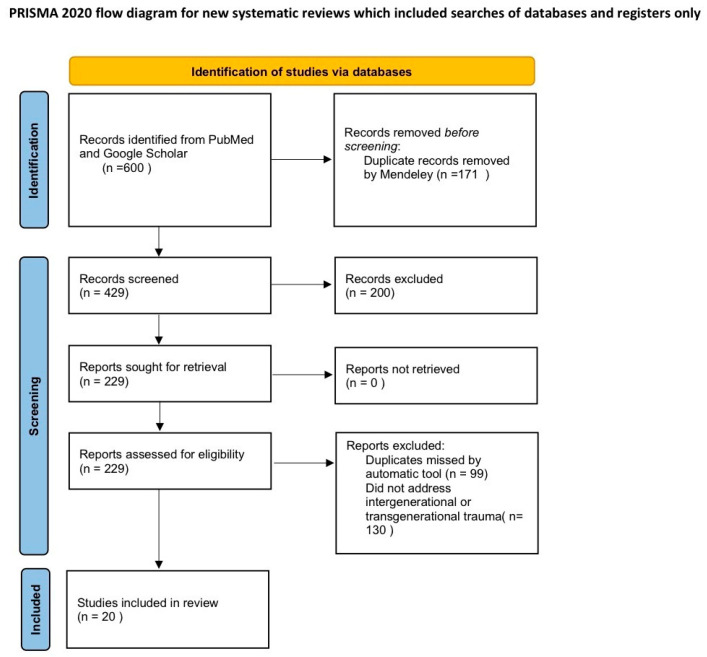
The flowchart of study search strategy.

The systematic review was conducted in accordance with PRISMA 2020 guidelines. The review was not registered in a prospective review registry. The PRISMA 2020 checklist can be found in the Supplementary Materials (Figure S1). To answer the question posed, we conducted searches of individual databases using the following keywords: trauma transgenerational (1), transgenerational trauma (2), transgenerational epigenetic (3), and transgenerational inheritance (4). A systematic search of PubMed and Google Scholar was conducted on 17 November 2025 covering relevant studies published over the last two decades, as they contained the most research. Full Search Strategy can be found in Supplementary Materials (Table S1). We included human empirical studies (interventional and observational) that assessed trauma exposure in a parental generation and measured biological (e.g., DNA methylation, cortisol) and/or psychological/relational outcomes in offspring. In addition, we used selected high-quality reviews to contextualize mechanisms but did not treat them as primary evidence. The database search yielded 600 records. After removal of duplicates using Mendeley, 429 records remained for title and abstract screening. Following screening, 200 records were excluded (e.g., non-original articles, non-human studies, non-English papers, or studies not related to psychological trauma), leaving 229 reports assessed for full-text eligibility. Of these, 209 were excluded with reasons (most commonly: absence of an intergenerational design or outcomes; inappropriate population; incorrect exposure or outcome measures; duplicate records or insufficient data). Ultimately, 20 studies met the inclusion criteria and were included in the qualitative synthesis (Figure 1—Flowchart) Two authors independently presented the following data collected from published articles: study characteristics (first author, year of publication, type of study), characteristics of transgenerational trauma (DNA methylation, source of trauma, and intergenerational links), and available data on the prevalence of mental disorders (unipolar affective disorders, number of patients, severity, duration). Risk of bias was assessed according to study design using validated, design-specific tools. Randomized controlled trials were evaluated using the Cochrane Risk of Bias 2 (RoB 2) tool (Table S2 in Supplementary Materials), which assesses bias arising from the randomization process, deviations from intended interventions, missing outcome data, measurement of outcomes, and selection of reported results. Each domain was rated as “low risk”, “some concerns”, or “high risk”, and an overall judgment was assigned. Non-randomized intervention studies were assessed using the Risk of Bias in Non-randomized Studies of Interventions (ROBINS-I) tool (Table S3 in Supplementary Materials). This instrument evaluates bias due to confounding, participant selection, classification of interventions, deviations from intended interventions, missing data, outcome measurement, and selective reporting. Overall risk of bias was categorized as low, moderate, serious, or critical. Observational studies (cohort, case–control, and cross-sectional designs) were evaluated using the Newcastle–Ottawa Scale (NOS) (Table S4 in Supplementary Materials) This scale assesses methodological quality across three domains: selection (maximum 4 stars), comparability (maximum 2 stars), and outcome/exposure assessment (maximum 3 stars), with a maximum score of 9 stars. Studies scoring 7–9 stars were considered high quality, 5–6 moderate quality, and ≤4 low quality. The level of evidence for each included study was classified according to the Oxford Centre for Evidence-Based Medicine (OCEBM) hierarchy. Individual randomized controlled trials were classified as Level 2 evidence, while prospective observational cohort studies were classified as Level 2 or 3 depending on study design and outcome assessment. Observational case–control and cross-sectional studies were classified as Level 3 or Level 4 evidence. Non-randomized intervention studies and quasi-experimental designs were classified as Level 3 evidence. Qualitative studies were classified as Level 5 evidence. Study protocols were not assigned a level of evidence, as they do not report empirical outcome data. The assessment of the Risk of Bias Assessment and Evidence Grading of Included Studies (NOS, RoB 2, ROBINS-I, OCEBM)) are presented in Table S5 in the Supplementary Materials.

## 3. Results

### 3.1. Biological and Molecular Mechanisms

Biological evidence suggests that vulnerability to PTSD may be transmitted across generations, primarily through maternal pathways. Proposed mechanisms include altered hypothalamic–pituitary–adrenal (HPA) axis programming and changes in gene expression influenced by maternal biology—such as elevated cortisol during pregnancy—as well as early-life effects of parental behavior on the child’s stress-response system. Other potential mechanisms, although beyond the scope of this paper, have also been suggested. These include genetic polymorphisms that lower the threshold for PTSD, the development of threat-focused cognitive schemas learned from parents with PTSD, and exposure to parents’ trauma narratives or imagery, which may increase vulnerability to later trauma and PTSD [16].

#### 3.1.1. Trauma in Epigenetics

A study published in 2014 by Yehuda et al. showed changes in the methylation of the *NR3C1 GR-1F*—glucocorticoid receptor gene promoter—in children of Holocaust survivors in relation to PTSD in their mothers and fathers. The study included adult children who had at least one parent who survived the Holocaust (n = 80) and participants with similar demographic characteristics whose parents did not experience the Holocaust or suffer from PTSD (n = 15). Children of Holocaust survivors have specific methylation patterns of the *NR3C1* gene that correlate with the sex of the parent. When the mother suffered from PTSD, her children showed higher levels of gene methylation, i.e., lower receptor sensitivity, which is associated with increased mood deterioration, depressive and anxiety disorders, and the onset of PTSD. In contrast, PTSD in the father was associated with lower methylation levels in his children. They showed a tendency toward an insecure attachment style and a predisposition to mental disorders. Three influences on the methylation of the glucocorticoid receptor gene promoter have been demonstrated: (1) A mother with PTSD has a superior function over the methylation of a father without PTSD, (2) A father with PTSD dominates methylation over a mother without PTSD, (3) Both parents with PTSD do not mutually reinforce the effect of methylation—PTSD in a mother with PTSD mitigates the effect of methylation in a father with PTSD, so the child of these parents will have lower methylation. There may be several sources for this third case (sperm in men), such as the egg cell, hormonal balance during pregnancy, and the postpartum period [5].

A randomized controlled study which examined associations between early adversity, a parent–infant psychotherapy intervention (IMH-HV), and DNA methylation in 73 mother–child dyads, with children aged 0–24 months at baseline. Assessments were conducted at two time points: pre-intervention and 12 months later. Saliva samples were collected from both mothers and children to assess DNA methylation at candidate loci, including *Brain-Derived Neurotrophic Factor (BDNF)*, *Solute Carrier Family 6 Member 4 (SLC6A4)*, *Nuclear Receptor Subfamily 3*, *Group C*, *Member 1 (NR3C1)*, and *long interspersed nuclear element-1 (LINE-1)*. In mothers, baseline DNA methylation levels at these loci were not significantly associated with maternal childhood adversity, current stress exposure, sociodemographic variables, or participation in the IMH-HV intervention. Maternal methylation patterns also showed no significant change over time attributable to the intervention. In contrast, children’s DNA methylation showed greater sensitivity to the caregiving environment. Infant *SLC6A4* and *LINE-1* methylation were associated with parenting attitudes and parent–child interaction quality. Importantly, *BDNF* methylation in infants decreased significantly over the 12-month period among those whose families participated in the psychotherapy intervention, suggesting an intervention-related biological effect. Overall, the study indicates that while maternal epigenetic profiles appeared relatively stable, early relational experiences and psychotherapy were associated with measurable epigenetic changes in infants, supporting the notion that early childhood represents a biologically sensitive window for intervention [8]. DNA methylation does not alter the genetic code itself-it leaves the DNA sequence unchanged. What it does instead is control how much a gene is used, deciding whether it is active, less active, or switched off. The easiest way to understand this is to think of it as a “biological volume knob”—the gene is still there, but its activity can be turned up or down depending on the situation. When the body experiences severe environmental stress, it may dial down genes involved in coping with stress. This can lead to long-lasting changes, where even future generations respond to stress differently, as if their baseline sensitivity has been reset.

#### 3.1.2. Endocrinology in Trauma

Yehuda et al. found that offspring of parents with PTSD exhibited lower cortisol levels on average. This does not indicate a lack of stress, but rather a hypersensitivity of the stress inhibitory system (HPA axis). Acute stress responses result in low cortisol levels and impaired ability to perform the adaptive “fight or flight” response. The study included 23 men and 26 women, all adult offspring of Holocaust survivors. Results indicate that parental PTSD, especially maternal PTSD, is linked to long-lasting alterations in HPA axis functioning in offspring. Individuals whose parents met criteria for PTSD exhibited significantly lower 24 h urinary cortisol levels, reflected in a reduced overall cortisol output (mesor) and a flattened diurnal rhythm, compared with offspring of trauma-exposed parents without PTSD and non-exposed controls. These differences were independent of the offspring’s own PTSD or other psychiatric disorders, suggesting that parental PTSD represents an independent biological vulnerability. The strongest associations were observed in cases of maternal PTSD, highlighting the possible influence of prenatal factors, early caregiving, or both on long-term stress regulation. Low cortisol levels indicate adjustment for a life of constant threat, which under normal circumstances becomes a burden. The authors further suggest that parental PTSD may affect offspring neuroendocrine development through biological programming mechanisms, such as altered in utero glucocorticoid exposure or early postnatal maternal behaviors. Additionally, lower cortisol levels were found in offspring born closer in time to the Holocaust, even after controlling for body mass index, age, and mood or anxiety disorders, indicating a temporal proximity effect of parental trauma on offspring stress physiology [4].

#### 3.1.3. Metabolomics and Aging

Another important aspect may be the role of acetyl-L-carnitine (LAC), which could potentially serve as a biomarker. Acetyl-L-carnitine has been suggested to act as an epigenetic modulator of brain plasticity and a promising therapeutic target for clinical phenotypes of depression associated with childhood trauma [17]. A study conducted by Nasca et al. [13] examined patients experiencing an episode of major depression. A decrease in LAC levels, but not free carnitine levels, was found in patients with major depressive episodes compared to healthy individuals of the same age and gender. Additional analyses showed that the degree of LAC deficiency affected both the severity and age of onset of major depressive episodes. Furthermore, these analyses showed that the decrease in LAC levels was greater in patients with a history of treatment-resistant depression (TRD), among whom traumatic childhood experiences, specifically a history of emotional neglect, as well as female gender, predicted a decrease in LAC levels [13]. Research conducted in 2023 by Katrinli et al. confirmed that epigenetic changes in DNA methylation can affect the expression of trauma-related genes on human health. PTSD is associated with accelerated aging. Whole-genome DNA methylation was examined in the blood of 140 war veterans (Illumina Methylation EPIC Bead Chip). 112 of them had a diagnosis of PTSD and 28 veterans had no diagnosis of PTSD. Patients with PTSD showed increased DNA methylation and accelerated GrimAge (epigenetic marker of mortality) compared to trauma-exposed and non-trauma-exposed individuals. Studies show that there is a possibility of higher risk of premature mortality in individuals with PTSD symptoms [14]. In the randomized controlled study of Ryan et al. they examined the effects of a family-based intervention on stress regulation, psychological functioning, and epigenetic outcomes in children of mothers (n = 62 women-child dyads) exposed to severe war-related trauma. Mothers and their children were assigned either to a family therapy program or to a waitlist control condition. Children in the intervention group showed a significant reduction in cortisol levels following treatment, suggesting improved regulation of physiological stress, whereas no such improvement was observed in the control group. Epigenome-wide analyses identified multiple differentially methylated CpG sites between groups, although none remained significant after correction for multiple testing. Importantly, the intervention did not influence epigenetic age acceleration, which remained strongly correlated with chronological age. Overall, the findings indicate that family-based therapeutic support can reduce biological stress in children from trauma-affected families, while evidence for lasting epigenetic modification remains preliminary and warrants further investigation [10].

### 3.2. Relational and Psychological Mechanisms

In the study by Zhou et al., evidence shows severe childhood abuse is associated with increased methylation of the gene coding for the glucocorticoid receptor (*NR3C1*) in the hippocampus which was found in post-mortem investigation of suicide victims. Similar associations have been found in adults reporting parental loss, insufficient care, or maltreatment, often accompanied by a blunted cortisol stress response. The most consistent evidence comes from studies of prenatal stress. Maternal stress during pregnancy—including perceived stress, anxiety, depression, and natural disasters—has been repeatedly linked to elevated *NR3C1* methylation in newborns across various tissues (e.g., cord blood, placenta, buccal cells). Increased *NR3C1* methylation has been observed both in infants exposed to general prenatal stress and in those born to mothers experiencing third-trimester depression or anxiety [15].

### 3.3. Three Generations

The “first” generation directly experiences traumatic events. A study by Brave Heart et al. in 2020 described the phenomenon of “historical trauma response” (HTR) as features of collective and intergenerational psychological responses, including unresolved grief and depressive symptoms, arising from cumulative historical trauma [1]. In turn, a 2019 study by Robjant et al. shows that first-generation individuals often exhibit severe symptoms of PTSD and psychological adaptation that lowers the threshold for aggressive behavior in response to stressful situations, reducing the chances of creating a safe environment for their future children [2]. The second generation is not directly exposed to trauma as the first generation was, yet it develops a kind of sensitivity. In a 2020 study, Hajal et al. describe that the perceived threat by parents during traumatic periods such as war or famine is a stronger factor in their child’s poorer adaptation than if they had experienced it in real time. The clinical manifestation in the second generation takes the form of chronic PTSD and a significant deficit in parental attentiveness. This inability to respond to their children’s needs can contribute to disorganized attachment disorders and hypervigilance in everyday life, passing on a sense of constant danger as anxiety disorders [9]. The complexity of trauma and symptoms of psychopathology can be observed in the third generation. A study by Gathier et al. from 2023 noted the occurrence of major depressive disorder (MDD) in adults with a history of childhood trauma in the family. These patients often have a weaker response to treatment and, as a result, treatment-resistant depression [7]. Furthermore, a 2022 study by Kaliman et al. highlights that third-generation adolescents who have experienced multiple adverse childhood experiences (ACEs) have noticeable epigenetic changes in the genes responsible for stress response. This generation experiences chronic anxiety and an inability to self-regulate, even in calm, everyday situations that are not life-threatening [12].

#### Clinical Manifestations Across Three Generations

Studies conducted in post-conflict and military settings indicate that transgenerational trauma manifests clinically in offspring across the lifespan, with symptom expression varying according to developmental stage. Burchert et al. (2017) [18] examined mothers directly exposed to the Khmer Rouge regime and their adult offspring born after the end of the conflict. The findings show that maternal lifetime traumatic exposure is associated with increased vulnerability to post-traumatic stress symptoms in offspring, with particularly pronounced effects observed in daughters. At comparable levels of trauma exposure, daughters exhibited higher PTSS/PTSD symptom scores than sons, suggesting sex-specific patterns in the clinical manifestation of transgenerational trauma [18]. In childhood, transgenerational trauma appears to be expressed less through discrete psychiatric syndromes and more through emotional and behavioral difficulties. In Seery et al. (2024) [19], children of mothers who experienced conflict-related sexual violence were described as presenting with emotional and behavioral difficulties despite not having been directly exposed to war-related trauma. The authors interpreted these difficulties as transgenerational trauma emerging within the caregiving and family context, reflecting disruptions in psychosocial functioning rather than discrete psychiatric diagnoses [19]. The same findings were reported by Hajal et al. (2020) [9], who identified clinically relevant manifestations of transgenerational trauma in children of fathers exposed to wartime deployment. Higher levels of deployment-related perceived threat reported by fathers were significantly associated with increased child behavior problems and higher overall levels of difficulties, reflecting impairments in behavioral regulation and psychosocial adjustment. Fathers’ PTSD symptoms were additionally related to elevated total difficulties scores in children. In contrast, child anxiety was not associated with fathers’ trauma-related deployment experiences, indicating that offspring manifestations were primarily expressed through behavioral and adjustment difficulties rather than anxiety-related symptoms. Sex differences were observed, with girls exhibiting lower overall difficulty scores than boys [9]. At the earliest stages of development, manifestations of transgenerational trauma are primarily observed at the level of regulation and relationship functioning. In Petroff et al. (2024) [8], infants from families affected by intergenerational social adversity were described as exhibiting regulatory difficulties, including heightened reactivity and reduced capacity for self-regulation. Further clinically significant problems were observed at the relational level, with altered parent-infant interaction patterns characterized by reduced emotional synchrony and difficulties in dyadic attunement [8]. In the research by Breton et al. evidence for transgenerational effects of stress and trauma on neurodevelopment—beyond direct parental exposure—remains limited and inconclusive. Most third-generation studies have focused on descendants of Holocaust survivors. A meta-analysis of 13 samples (1012 participants) found no evidence of tertiary traumatization, with grandchildren of survivors showing similar psychological well-being and adaptation as comparison groups. These findings suggest that Holocaust survivors may represent a particularly resilient group, potentially contributing to positive outcomes in subsequent generations. Outside the Holocaust literature, one study of over 2200 first-grade children in Shanghai reported more emotional and behavioral problems among children whose parents or grandparents experienced trauma—but only in parent reports. Teacher reports did not replicate this pattern, and associations largely disappeared after adjusting for parental mental and physical health, indicating possible reporting bias. Taken together, available studies for third-generation effects of trauma on neurodevelopment are inconsistent. Studies remain few, methodologically heterogeneous, and often rely on retrospective or proxy reports that may introduce bias [20]. Characteristics of Randomized Controlled Trials (RCTs) evaluating interventions for transgenerational trauma 2005–2025 (Table 1) and characteristics of non-randomized clinical trials and observational studies included in the review 2005–2025 (Table 2) are below.

## 4. Discussion

Studies by Yehuda et al. from 2007 and 2014 show that low cortisol levels in children who survived the Holocaust may be a marker of transgenerational trauma [4,5]. We also note that changes in the hypothalamic–pituitary–adrenal (HPA) axis are associated with local DNA methylation (DNAm) of the *NR3C1* gene. Gender also plays a role, depending on whether PTSD occurred in the mother or father. Therefore, trauma transmission occurs through different biological pathways responsible for stress responses before offspring encounter direct threat. Studies by Ryan et al. from 2024 show that molecular traces are detectable in the third generation [10]. The main conclusion of our review is that we cannot fully confirm the three-generation hypothesis. The impact of trauma is not only psychological but also biological. Animal studies allow for rigorous control of variables, whereas in humans, the inheritance of trauma is a combination of both biological and environmental factors. It is impossible to definitively determine whether a child inherits anxiety genetically or learns it from observing an overprotective mother. These two mechanisms likely feed into each other. Caution should be exercised in interpreting the results. Further research is needed, however, to confirm that trauma experienced by the first generation has a cascading effect, with the stressful environment of the mother or father (the second generation) acting as a conduit for epigenetic imprints in their parents (the third generation). Although biological evidence provides a solid explanation for the transmission of trauma, a psychological aspect is still needed to explain the complexity of intergenerational transmission. Caregivers’ attentiveness to responding appropriately to a child’s needs is a key psychological mediator that can either strengthen or weaken inherited epigenetic susceptibility. Parents exposed to trauma (second generation) often show impaired responses to their children’s needs, as shown in a 2022 study by Condon et al. [23]. A parent who has not worked through their own traumatic events from their past. The child’s hereditary sensitivity to *NR3C1* methylation, combined with their parent’s inability to respond, prevents the child from developing a healthy stress response system, effectively perpetuating the epigenetic marks inherited from the first generation. The effectiveness of relational interventions studied by Zayde et al. in 2023 and the “Minding the Baby” program points to epigenetic plasticity [27]. The interventions focus on strengthening parents’ mindfulness and responsiveness to their children. The parent acts as a protective shield that can mitigate or even stop the effects of inherited trauma. Psychotherapy has been shown to provide not only psychological support, but also changes in further methylation. As shown by the results of research by Petroff et al. in 2024, the quality of intervention can lead to long-term changes in DNA methylation, resulting in improved parent–child bonding as a fundamental mechanism for interrupting the intergenerational transmission of trauma [8].

Although evidence of traumatic experiences, such as the acceleration of the epigenetic clock (GrimAge) in individuals with PTSD demonstrated in the 2023 study by Katrinli et al. and metabolic deficits from the 2018 study by Nasca et al. note negative effects on the third generation, recent longitudinal studies indicate strong counterarguments to the persistence and configuration of these effects [13,14]. The results of a study by Carleial et al. from 2021 show that narrative exposure therapy (NET) can cause significant changes in DNA methylation patterns in people exposed to trauma [25]. Similarly, a 2022 study by Kaliman et al. showed that even short-term interventions with third-generation adolescents who had difficult childhood experiences can affect the genes that regulate stress response [12].

The main advantage of our systematic review is a broad analysis of research on intergenerational trauma, covering both molecular biology and clinical psychology. Metabolomics, such as LAC deficiency and epigenetic markers, namely *NR3C1* methylation and GrimAge acceleration with clinical manifestations of intergenerational trauma, reveal the complexity of trauma transmission. Unlike previous reviews, which focused mainly on psychological narratives, our approach provides a biological rationale for the three-generation hypothesis: grandparents-parents-grandchildren. Taking into account all randomized controlled trials (RCTs) on trauma transmission and demonstrating epigenetic plasticity, it emphasizes the intergenerational effect and offers hope for the potential reversal of these effects through psychotherapeutic interventions.

Despite the evidence cited in this article, its limitations should be kept in mind. Many of the included studies, particularly the 2007 and 2014 studies by Yehuda et al. and the 2019 study by Herbell, were based on small samples, resulting in limited statistical power and limited generalisability of the findings [4,5,25]. There is also considerable heterogeneity in the types of traumas analyzed, namely large-scale historical and war trauma, individual intimate partner violence, and childbirth-related PTSD. Separating environmental from genetic influences in trauma is challenging. This diversity of traumatic experiences makes it difficult to establish a single, general marker for all transgenerational traumas. The relationship between genetic inheritance and environmental influences remains a significant methodological challenge. Despite evidence from epigenetic markers suggesting a molecular approach, the simultaneous influence of attachment disorders as insecure attachment styles is complex and difficult to isolate. There is also a potential risk of inaccurate memory or significant memory lapses, as well as unintentional misinterpretations of past events, which can impact the quality of intergenerational bonds. The aim of the study was not to compare these traumas but to examine whether there is a common trauma gene regardless of the source of the stress.

## 5. Conclusions

Taken together, available studies suggest that parental trauma—particularly maternal PTSD—may have long-lasting biological consequences for offspring, mainly through dysregulation of the HPA axis and altered stress physiology. Children of trauma-exposed parents often exhibit lower cortisol levels, impairing stress responses. Family- and relationship-based psychotherapeutic interventions have been shown to reduce physiological stress in children, as reflected by posttreatment cortisol levels decreasing to within physiological limits. Early childhood appears to be a biologically sensitive window during which psychosocial interventions can shape stress regulation and epigenetic processes. During this stage, the epigenome shows greater plasticity, and psychotherapy has been associated with gene-specific changes in DNA methylation in pathways involved in stress regulation and neurodevelopment, including BDNF and SLC6A4. Although evidence for broad, genome-wide epigenetic reprogramming remains limited, these targeted findings support the concept of biological embedding of experience. In summary, current evidence suggests that psychotherapy may work not only at the psychological level, but also at the biological level, affecting physiology and epigenetic regulation, thereby reducing the transgenerational transmission of trauma susceptibility.

## Figures and Tables

**Table 1:** Characteristics of Randomized Controlled Trials (RCTs) evaluating interventions for transgenerational trauma 2005–2025.

Author	Journal	Title	Year of Publication	Group	Main Conclusion
Brave Heart et al. [1]	Psychotherapy	Iwankapiya American Indian pilot clinical trial: Historical trauma and group interpersonal psychotherapy	2020	n = 52 American Indian adults	Healing is transgenerational and nonlinear, linking present experiences with ancestral suffering. Iwankapiya Group Interpersonal Psychotherapy combined with historical trauma and unresolved grief reduces trauma, depression, and grief symptoms. Transgenerational trauma involves accumulated grief, guilt, and low self-esteem rooted in internalized historical oppression. Affected individuals show heightened sensitivity to contemporary stressors.
Robjant et al. [2]	Behaviour Research and Therapy	The treatment of posttraumatic stress symptoms and aggression in female former child soldiers using adapted Narrative Exposure therapy—a RCT in Eastern Democratic Republic of Congo	2019	n = 92 female former child soldiers	Transgenerational trauma in war-affected families affects dysregulation, with violence used as a coping strategy. Interrupting trauma transmission requires addressing perpetration, guilt, and violent acts. Appetitive aggression sustains post-conflict violence, may protect against PTSD, but reduces motivation for treatment.
Gathier et al. [7]	BMC Psychiatry	Design and rationale of the REStoring mood after early life trauma with psychotherapy (RESET-psychotherapy) study: a multicenter randomized controlled trial on the efficacy of adjunctive trauma-focused therapy (TFT) versus treatment as usual (TAU) for adult patients with major depressive disorder (MDD) and childhood trauma	2023	n = 158 adults with moderate to severe major depressive disorder	Childhood trauma changes the way the patient responds to severe depression therapy, unconscious trauma influences the symptoms of depression. The therapist treats only the effect and not the traumatic source (from parents), the cycle of suffering remains active—masking the trauma with depression.
Petroff et al. [8]	Brain and Behavior	Longitudinal DNA methylation in parent–infant pairs impacted by intergenerational social adversity: An RCT of the Michigan Model of Infant Mental Health Home Visiting	2024	n = 146, 73 pairs-mothers and their infants/toddlers between 0 and 24 months	Specifically, higher scores on a mother-report measure of child abuse potential were inversely associated with *SLC6A4* methylation.
Hajal et al. [9]	Journal of Traumatic Stress	Parental Wartime Deployment and Socioemotional Adjustment in Early Childhood: The Critical Role of Military Parents’ Perceived Threat During Deployment	2020	n = 104 of military-connected families	The strong perception of a soldier’s life being threatened is a source of problems for children after returning from war. Secondary trauma, because the parent, who may have died, is constantly under stress, impacting the family atmosphere and the child’s sense of security. If the parent feels threatened, the child will not feel safe.
Ryan et al. [10]	Brain and Behavior	An epigenome-wide study of a needs-based family intervention for offspring of trauma-exposed mothers in Kosovo	2024	n = 62 women-child dyads	Maternal exposure to trauma is associated with epigenetic alterations in children, indicating intergenerational biological effects. Identified epigenetic changes were enriched in immune-related genes, suggesting a pathway linking transgenerational trauma to somatic disease risk in adulthood. The findings support the concept of biological embedding of experience, whereby maternal trauma is reflected in the DNA methylation patterns of offspring. Psychotherapy functions as a biological intervention, capable of modifying gene methylation in pathways relevant to offspring mental health.
Kaliman et al. [12]	Scientific Reports	Epigenetic impact of a 1-week intensive multimodal group program for adolescents with multiple adverse childhood experiences	2022	n = 44	Even a very brief but intense stressful event can trigger measurable epigenetic changes in traumatized youth. DNA methylation changes at 657 CpG sites after just seven days. These changes are crucial for immune response, neural plasticity, and emotion regulation.
Burchert et al. [18]	Psychiatry Research	Transgenerational trauma in a post-conflict setting: Effects on offspring PTSS/PTSD and offspring vulnerability in Cambodian families	2017	n = 378 mothers and their children	No direct intergenerational transmission of maternal trauma, PTSS, or PTSD to offspring was found. Maternal trauma increases vulnerability, not psychopathology per se, which may manifest only after offspring experience their own stressors. A sex-specific moderating effect was observed. Maternal hyperarousal was the only PTSS symptom associated with offspring PTSS. Closer mother-daughter relationships may increase daughters’ sensitivity to maternal trauma and reduce their ability to cope with their own traumatic experiences.
Seery et al. [19]	Clinical Psychology & Psychotherapy	Family Therapy for Kosovar Mothers Who Experienced Conflict-Related Sexual Violence and Their Children in Postwar Times: A Pilot Randomised Waitlist-Controlled Trial	2024	n = 64 mothers and their children	The mechanism of trauma transmission is systemic, not individual. The intra-family relationship acts as a moderator that supports child development, inhibiting the intergenerational transmission of anxiety. Children in family therapy showed improved mental health.
Hill et al. [21]	Journal of Women’s Health	Trauma-Informed Personalized Scripts to Address Partner Violence and Reproductive Coercion: Preliminary Findings from an Implementation Randomized Controlled Trial	2019	n = 240 participants English-speaking females, ages 16–29	Intimate partner violence and reproductive coercion are critical but often overlooked forms of trauma. Transgenerational trauma may begin with the loss of reproductive autonomy, affecting the next generation from conception onward. Breaking the intergenerational transmission of trauma requires a trauma-informed care system that identifies and intervenes in violent relationship patterns early, before they become normalized for children.
Carleial et al. [22]	Scientific Reports volume	DNA methylation changes following narrative exposure therapy in a randomized controlled trial with female former child soldiers	2021	n = 84 female former child soldiers from Eastern DR Congo	Effective narrative psychotherapy for Narrative Exposure Therapy leads to measurable changes in DNA methylation. Thirty-three CpG methylation regions were identified, and DNA methylation levels significantly changed after NET compared to the control group.
Condon et al. [23]	Child Maltreatment	Examining Mothers’ Childhood Maltreatment History, Parental Reflective Functioning, and the Long-Term Effects of the Minding the Baby^®^ Home Visiting Intervention	2022	n = 97	Childhood maltreatment of women (mothers) is strongly linked to lower levels of parental reflective function, along with a failure to recognize the emotional needs of their own child. A mother who lacks a secure attachment is unable to bond with her child, and the trauma is passed on to the next generation, often developing a disorganized attachment style in the infant. A mother’s anxiety and emotional instability become a permanent feature of the child’s developmental environment.
Devita et al. [24]	Journal of Reproductive and Infant Psychology	Maternal childbirth-related posttraumatic stress symptoms, bonding, and infant development: a prospective study	2025	n = 55 mother–infant dyads	The severity of PTSD symptoms after childbirth is directly linked to insecure attachment styles with the child after birth—emotional distance, poor interactions with the child, and a lack of positive moments.
Herbell et al. [25]	Child Abuse & Neglect	Keeping it together for the kids: New mothers’ descriptions of the impact of intimate partner violence on parenting.	2020	n = 11 women	Maternal domestic trauma alters DNA methylation in newborns. Mothers’ hypervigilance—their attention is divided between the child’s needs and monitoring the abuser’s mood. In children, transgenerational trauma begins with growing up in an atmosphere of unspoken tension, where the child senses, for example, their mother’s anxiety, even if they do not witness acts of violence.

**Table 2:** Characteristics of non-randomized clinical trials and observational studies included in the review 2005–2025.

Author	Title	IF (Year)	Group	Type	Main Conclusion
Yehuda et al. [4]	Parental posttraumatic stress disorder as a vulnerability factor for low cortisol trait in offspring of holocaust survivors	10.78 (2007)	23 Holocaust offspring with parental PTSD and 10 without parental PTSD compared with 16 children of nonexposed parents. n = 49	Comparative Study	Biological markers of trauma are inherited by offspring, even if they themselves have not experienced direct trauma. Free cortisol levels in children of Holocaust survivors, children of parents with PTSD, have lower cortisol levels compared to the control group. Low cortisol levels in children were directly linked to the presence of PTSD in the parent (especially the mother), not to the mere fact of being a survivor. Living with constant threat results in low cortisol levels, which increases hyperreactivity to external stimuli and an inability to cope with them.
Yehuda et al. [5]	Influences of maternal and paternal PTSD on epigenetic regulation of the glucocorticoid receptor gene in Holocaust survivor offspring	12.2 (2014)	Adult offspring with at least one Holocaust survivor parent (n = 80) and demographically similar participants without parental Holocaust exposure or parental PTSD (n = 15)	Cross-sectional Study	The NR3C1 gene promoter (region 1F) is responsible for regulating stress sensitivity via the glucocorticoid receptor. Children of Holocaust survivors have specific methylation patterns of this gene, depending on the gender of their parent: PTSD in the mother—higher methylation of the gene in children, which indicates lower receptor sensitivity and a greater likelihood of depression, anxiety, and low mood. PTSD in the father was associated with lower methylation in children, which indicates a predisposition to an anxious attachment style and insecurity.
Nasca et al. [13]	Acetyl-l-carnitine deficiency in patients with major depressive disorder	9.58 (2018)	n = 116	Clinical trial	Patients with major depressive disorder have significantly lower levels of acetyl-l-carnitine (LAC) in their blood compared to healthy individuals, and the lowest levels are found in those who also experienced childhood trauma. LAC is crucial for the epigenetic regulation of brain plasticity. A deficiency can cause trauma to prevent the brain from adapting and regenerating.
Marsh et al. [26]	A study protocol for a quasi-experimental community trial evaluating the integration of indigenous healing practices and a harm reduction approach with principles of seeking safety in an indigenous residential treatment program in Northern Ontario	4.45 (2021)	2013–2016; n = 343; 2018–2020; n > 300	pre/post Quasi Experimental Community trial	The mechanism of trauma transmission is dysfunctional ways of regulating emotions (e.g., substance abuse) from parent to child as a learned model of escaping emotions.
Zayde et al. [27]	Safe haven in adolescence: Improving parental reflective functioning and youth attachment and mental health with the Connecting and Reflecting Experience	2.4 (2023)	n = 32 caregiver-adolescent dyads	Clinical Trial	A parent’s trauma often impairs their reflective function. Parents are unable to read their child’s emotions, and the child reacts with anxiety or aggression, which is a primary channel for the transmission of transgenerational trauma. Withdrawal, self-aggression, and anxiety are directly linked to the parent’s lack of response, as they have their own unresolved traumatic experiences. Transgenerational trauma integrates molecular mechanisms (DNA methylation), psychological mechanisms (weakened parental reflective functions), and social mechanisms (history and violence).

## Data Availability

No new data were created or analyzed in this study.

## Supplementary Materials

The following supporting information can be downloaded at: https://www.mdpi.com/article/10.3390/cells15070609/s1; Figure S1: PRISMA 2020 Checklist [28]; Table S1: Search Strategy; Table S2: Cochrane Risk of Bias 2 (RoB 2); Table S3: Risk Of Bias In Non-randomized Studies of Interventions—ROBINS-I; Table S4: Newcastle–Ottawa Scale (NOS); Table S5: Risk of Bias Assessment and Evidence Grading of Included Studies (NOS, RoB 2, ROBINS-I, OCEBM).

## Abbreviations

The following abbreviations are used in this manuscript:

ACEs	Adverse Childhood Experiences
BDNF	Brain-Derived Neurotrophic Factor
CpG	Cytosine-guanine dinucleotide sites
CPTSD	Complex Post-Traumatic Stress Disorder
DNAm	DNA Methylation
HPA	Hypothalamic–Pituitary–Adrenal
HTR	Historical Trauma Response
LAC	Acetyl-L-carnitine
MDD	Major Depressive Disorder
NET	Narrative Exposure Therapy
NR3C1	Nuclear Receptor Subfamily 3 Group C Member 1 (Glucocorticoid receptor gene)
PRF	Parental Reflective Functioning
PTSD	Post-Traumatic Stress Disorder
SLC6A4	Solute Carrier Family 6 Member 4 (Serotonin transporter gene)
TRD	Treatment-Resistant Depression
GR	Glucocorticoid Receptor
IMH-HV	Infant Mental Health Home Visiting
LINE-1	Long Interspersed Nuclear Element-1
PTSS	Post-Traumatic Stress Symptoms
RCT	Randomized Controlled Trial
