# Supplementary material for: When Trauma Crosses Generations: Mechanisms, Clinical Patterns and Therapeutic Implications of Transgenerational Trauma—A Systematic Review
Source: Cells. 2026 Mar 30;15(7):609. doi: 10.3390/cells15070609 (PMC13072029; doi:10.3390/cells15070609)
Supplement: Supplementary file 1 [file cells-15-00609-s001.zip › Table S1. Search Strategy.pdf]

Table S1. Search Strategy

(("transgenerational trauma" OR "intergenerational trauma" OR "historical trauma"

OR "parental trauma" OR "parental PTSD" OR "maternal stress" OR "prenatal stress")

AND

("DNA methylation" OR epigenetic* OR "NR3C1" OR cortisol OR "HPA axis"

OR "stress response" OR "epigenetic aging" OR GrimAge)

AND

(offspring OR children OR adolescents OR adults))

Filters: Humans, English, 2005–2025..
