# Supplementary material for: When Trauma Crosses Generations: Mechanisms, Clinical Patterns and Therapeutic Implications of Transgenerational Trauma—A Systematic Review
Source: Cells. 2026 Mar 30;15(7):609. doi: 10.3390/cells15070609 (PMC13072029; doi:10.3390/cells15070609)
Supplement: Supplementary file 1 [file cells-15-00609-s001.zip › Table S2. Cochrane Risk of Bias 2 (RoB 2).pdf]

Table S2. Cochrane Risk of Bias 2 (RoB 2)

Study ID	Title	D1	D2	D3	D4	D5	Overall
Burchert et al. ; 2017	Transgenerational trauma in a post-conflict setting: Effects on offspring PTSS/PTSD and offspring vulnerability in Cambodian families	High risk	High risk	Some concerns	Some concerns	High risk	High risk
Hill et al.; 2019	Trauma-Informed Personalized Scripts to Address Partner Violence and Reproductive Coercion: Preliminary Findings from an Implementation Randomized Controlled Trial	Some concerns	Some concerns	Low risk	Some concerns	Some concerns	Some concerns
Robjant et al., 2019	The treatment of posttraumatic stress symptoms and aggression in female former child soldiers using adapted Narrative Exposure therapy - a RCT in Eastern Democratic Republic of Congo	Some concerns	Some concerns	Some concerns	Some concerns	Some concerns	Some concerns
Brave Heart et al.; 2020	Iwankapiya American Indian pilot clinical trial: Historical trauma and group interpersonal psychotherapy	Some concerns	Some concerns	Some concerns	Some concerns	Some concerns	Some concerns
Herbell et al.; 2020	Keeping it together for the	High risk	High risk	High risk	High risk	High risk	High risk

	kids: New mothers' descriptions of the impact of intimate partner violence on parenting						
Hajal et al.; 2020	Parental Wartime Deployment and Socioemotional Adjustment in Early Childhood: The Critical Role of Military Parents' Perceived Threat During Deployment	High risk	High risk	Some concerns	Some concerns	High risk	High risk
Carleial et al.; 2021	DNA methylation changes following narrative exposure therapy in a randomized controlled trial with female former child soldiers	Some concerns	Some concerns	Some concerns	Low risk	Some concerns	Some concerns
Condon et al. 2022	Examining Mothers' Childhood Maltreatment History, Parental Reflective Functioning, and the Long-Term Effects of the Minding the Baby® Home Visiting Intervention	Some concerns	Some concerns	Some concerns	Some concerns	Some concerns	Some concerns
Kaliman et al.; 2022	Epigenetic impact of a 1-week intensive multimodal group program for adolescents with multiple	Some concerns	Some concerns	Low risk	Low risk	Some concerns	Some concerns

	adverse childhood experiences						
Gathier et al. 2023	Design and rationale of the REStoring mood after early life trauma with psychotherapy (RESET-psychotherapy) study: a multicenter randomized controlled trial on the efficacy of adjunctive trauma-focused therapy (TFT) versus treatment as usual (TAU) for adult patients with major depressive disorder (MDD) and childhood trauma	High risk	High risk	High risk	High risk	High risk	High risk
Seery et al.; 2024	Family Therapy for Kosovar Mothers Who Experienced Conflict-Related Sexual Violence and Their Children in Postwar Times: A Pilot Randomised Waitlist-Controlled Trial	Some concerns	Some concerns	Some concerns	Some concerns	Some concerns	Some concerns
Ryan et al.; 2024	An epigenome-wide study of a needs-based family intervention for offspring of trauma-exposed mothers in Kosovo	Some concerns	Some concerns	Low risk	Some concerns	Some concerns	Some concerns
Petroff et al.; 2024	Longitudinal DNA	Some concerns	Some concerns	Low risk	Some concerns	Some concerns	Some concerns

	methylation in parent–infant pairs impacted by intergenerational social adversity: An RCT of the Michigan Model of Infant Mental Health Home Visiting						
Devita et al.; 2025	Maternal childbirth-related posttraumatic stress symptoms, bonding, and infant development: a prospective study	High risk	High risk	High risk	High risk	High risk	High risk
