# Supplementary material for: When Trauma Crosses Generations: Mechanisms, Clinical Patterns and Therapeutic Implications of Transgenerational Trauma—A Systematic Review
Source: Cells. 2026 Mar 30;15(7):609. doi: 10.3390/cells15070609 (PMC13072029; doi:10.3390/cells15070609)
Supplement: Supplementary file 1 [file cells-15-00609-s001.zip › Table S3. Risk Of Bias In Non-randomized Studies of Interventions - ROBINS-I.pdf]

Table S3. Risk Of Bias In Non-randomized Studies of Interventions - ROBINS-I

Study ID	Title	Type	D1	D2	D3	D4	D5	D6	D7	Overall
Marsh et al; 2021	A study protocol for a quasi-experimental community trial evaluating the integration of indigenous healing practices and a harm reduction approach with principles of seeking safety in an indigenous residential treatment program in Northern Ontario	Quasi-experimental pre–post community study	Serious risk	Moderate risk	Moderate risk	Low risk	Moderate risk	Moderate risk	Serious risk	Serious risk
Zayde et al; 2023	Safe haven in adolescence: Improving parental reflective functioning and youth attachment and mental health with the Connectin	Clinical Trial	Serious risk	Moderate risk	Low risk	Moderate risk	Moderate risk	Serious risk	Serious risk	Serious risk

	g and Reflecting Experience
