# Supplementary material for: When Trauma Crosses Generations: Mechanisms, Clinical Patterns and Therapeutic Implications of Transgenerational Trauma—A Systematic Review
Source: Cells. 2026 Mar 30;15(7):609. doi: 10.3390/cells15070609 (PMC13072029; doi:10.3390/cells15070609)
Supplement: Supplementary file 1 [file cells-15-00609-s001.zip › Table S4. Newcastle–Ottawa Scale (NOS).pdf]

Table S4. Newcastle–Ottawa Scale (NOS)

Stydy ID	Type	Selection (4)	Comparability(2)	Outcome/Exposure (3)	Total (9)
Yehuda et al.; 2007	Observational case–control study	***	**	***	8/9
Nasca et al; 2018	Observational case–control study	***	**	***	8/9
Yehuda et al; 2014	Cross-sectional Study	****	**	***	9/9
Devita et al; 2025	Prospective Observational Cohort study	****	**	***	9/9
