# Supplementary material for: When Trauma Crosses Generations: Mechanisms, Clinical Patterns and Therapeutic Implications of Transgenerational Trauma—A Systematic Review
Source: Cells. 2026 Mar 30;15(7):609. doi: 10.3390/cells15070609 (PMC13072029; doi:10.3390/cells15070609)
Supplement: Supplementary file 1 [file cells-15-00609-s001.zip › Table S5 Risk of Bias Assessment and Evidence Grading of Included Studies (NOS, RoB 2, ROBINS-I, OCEBM).pdf]

Table S5: Risk of Bias Assessment and Evidence Grading of Included Studies (NOS, RoB 2, ROBINS-I, OCEBM).

Author	Title	Type	Category of evidence	Risk of Bias
Burchert et al.	Transgenerational trauma in a post-conflict setting: Effects on offspring PTSS/PTSD and offspring vulnerability in Cambodian families	Randomized Controlled Trial	Level II	High risk
Hill et al.	Trauma-Informed Personalized Scripts to Address Partner Violence and Reproductive Coercion: Preliminary Findings from an Implementation Randomized Controlled Trial	Randomized Controlled Trial	Level II	Some concerns
Robjant et al.	The treatment of posttraumatic stress symptoms and aggression in female former child soldiers using adapted Narrative Exposure therapy - a RCT in Eastern Democratic Republic of Congo	Randomized Controlled Trial	Level II	Some concers
Brave Heart et al.	Iwankapiya American Indian pilot clinical trial: Historical trauma and group interpersonal psychotherapy	Randomized Controlled Trial	Level II	Some concers
Herbell et al.	Keeping it together for the kids: New mothers' descriptions of the impact of intimate partner violence on parenting	Randomized Controlled Trial	Level II	High risk
Hajal et al.	Parental Wartime Deployment and Socioemotional Adjustment in Early Childhood: The Critical Role of Military Parents' Perceived Threat During Deployment	Randomized Controlled Trial	Level II	High risk
Carleial et al.	DNA methylation changes following narrative exposure therapy in a randomized controlled trial with female former child soldiers	Randomized Controlled Trial	Level II	Some concerns
Condon et al.	Examining Mothers' Childhood Maltreatment	Randomized Controlled Trial	Level II	Some concerns

	History, Parental Reflective Functioning, and the Long-Term Effects of the Minding the Baby® Home Visiting Intervention			
Kaliman et al.	Epigenetic impact of a 1-week intensive multimodal group program for adolescents with multiple adverse childhood experiences	Randomized Controlled Trial	Level II	Some concerns
Gathier et al.	Design and rationale of the REStoring mood after early life trauma with psychotherapy (RESET-psychotherapy) study: a multicenter randomized controlled trial on the efficacy of adjunctive trauma-focused therapy (TFT) versus treatment as usual (TAU) for adult patients with major depressive disorder (MDD) and childhood trauma	Randomized Controlled Trial	Level II	High risk
Seery et al.	Family Therapy for Kosovar Mothers Who Experienced Conflict-Related Sexual Violence and Their Children in Postwar Times: A Pilot Randomised Waitlist-Controlled Trial	Randomized Controlled Trial	Level II	Some concerns
Ryan et al.	An epigenome-wide study of a needs-based family intervention for offspring of trauma-exposed mothers in Kosovo	Randomized Controlled Trial	Level II	Some concerns
Petroff et al.	Longitudinal DNA methylation in parent–infant pairs impacted by intergenerational social adversity: An RCT of the Michigan Model of Infant Mental Health Home Visiting	Randomized Controlled Trial	Level II	Some concerns
Devita et al.	Maternal childbirth-related posttraumatic stress symptoms, bonding, and infant development: a prospective study	Randomized Controlled Trial	Level II	High risk

Author	Title	Year	Newcastle-Ottawa Scale	Category of evidence	Risk of Bias
Yehuda et al.	Parental posttraumatic stress disorder as a vulnerability factor for low cortisol trait in offspring of holocaust survivors	2007	Comparative Study	8/9	Level III
Marsh et al.	A study protocol for a quasi-experimental community trial evaluating the integration of indigenous healing practices and a harm reduction approach with principles of seeking safety in an indigenous residential treatment program in Northern Ontario	2021	pre/post Quasi Experimental Community trial	-	Not applicable
Nasca et al.	Acetyl-l-carnitine deficiency in patients with major depressive disorder	2018	Clinical trial	8/9	Level III
Yehuda et al.	Influences of maternal and paternal PTSD on epigenetic regulation of the glucocorticoid receptor gene in Holocaust survivor offspring	2014	Cross-sectional Study	9/9	Level III
